# Sensitivity Analysis of the Integral Quality Monitoring System® Using Monte Carlo Simulation

**DOI:** 10.1155/2017/7025281

**Published:** 2017-08-27

**Authors:** Oluwaseyi M. Oderinde, F. C. P. du Plessis

**Affiliations:** Department of Medical Physics, University of the Free State, P.O. Box 339, Bloemfontein 9300, South Africa

## Abstract

The Integral Quality Monitoring (IQM) System is a real-time beam output verifying system that validates the integrity and accuracy of patient treatment plan (TP) data during radiation treatment. The purpose of this study was to evaluate the sensitivity of the IQM to errors in segment using EGSnrc/BEAMnrc Monte Carlo (MC) codes. Sensitivity analysis (SA) techniques were applied to study the significance of small alterations of field sizes (segments) on the IQM signal response. One hundred and eighty multileaf segments were analyzed with methods that include scatter plots (SP), brute force, variance-based (VAR), and standard regression coefficient SA. The segments were altered randomly within ±1, ±2, and ±3 mm leaf steps for 10 MV photon beams. SP analysis gradient and VAR maximum index are 1.045 and 0.556 for the smallest segment while the largest segment has the value of 0.018 and 0.504, respectively. The brute force and standard regression displayed maximum sensitivity indices around the unaltered segments. These tests conclusively indicated that the IQM was more sensitive to alterations of small segments compared to larger segments. This is important since small segment variation will cause a higher dose output variation that should be picked up during online beam monitoring.

## 1. Introduction

The goal of radiotherapy is to improve the quality of treatment: minimizing the normal tissue exposure and maximizing the therapeutic ratio. In the quest for an optimum treatment, Paliwal introduced the concept of an online beam delivery check for noncomputerized Linacs [1]. It consisted of a transmission chamber to detect possible errors in treatment delivery and to serve as a pretreatment quality assurance (QA) tool. This concept adds an additional record-and-verify system to the Linac head. Since then, vendors have developed and suggested some dose monitors for offline/online dose verification of external photon beam radiotherapy. The suggested online monitors are the electronic portal imaging device (EPID), DAVID™, and the Dolphin® system [2–4].

**Figure 1:**
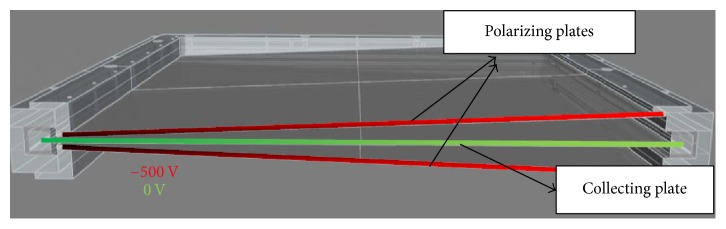
IQM double wedge ionization chamber model (*permission granted*) [27].

The Integral Quality Monitoring (IQM) System is a prototype online dose verification system that was released by iRT Systems, Germany. The IQM system is a double wedge-shaped ionization chamber that is attached below the Linac treatment head. It is a dose measuring system that validates the TP data in real time [5]. The IQM is capable of monitoring a 40 × 40 cm^2^ field defined at the isocenter. The double wedge shapes of the IQM device are defined by the outer polarizing electrodes which are kept at a potential of 500 volts. The inner electrode plate is grounded to zero volts and is designated as a collecting electrode (Figure 1). The electrodes are made of 1.5 mm aluminum. The output signal is a function of the photon beam fluence that irradiates the double-wedged chamber.

The major interest of utilizing the prototype IQM device is its ability to function as a beam delivery check system during real-time treatment [6, 7].

Sensitivity analysis is the process of undertaking a systematic review of models; it studies the significance of each of the model inputs on the model output [8, 9]. It identifies and determines the impact of inputs on its outputs [10–15]. This builds a theory that relates the input variables with the output variables. There are several sensitivity analysis techniques stated in various articles such as scatter plots, one-at-a-time, partial derivatives, brute force, partial correlation coefficient, standard regression coefficient, variance-based SA, Sobol sensitivity indices, fast first-order index, Spearman rank correlation coefficient, Fourier amplitude sensitivity test, and Morris one-at-a-time screening [10, 16–23]. The sensitivity ranking of the input for several techniques may vary slightly, but the focus is based on the consistent parameter that influences the output [13, 19, 24]. The choice of method of sensitivity analysis is guided by the problem constraints. The constraint can be correlated inputs, nonlinearity, multiple outputs, given data, or random variables (e.g., simple random sampling, Monte Carlo, Latin Hypercube, Morris method, and quasi-random sequence). Random sampling, which is the focus of this research, requires the application of scatter plots, brute force, variance-based, and standard regression coefficient sensitivity analysis techniques which are efficiently independent of one another [14, 16, 18, 21, 25, 26]. These four sensitivity analysis techniques were used for an in-depth study of the consistent input parameters that influenced the output value.

The aim of this study was to investigate the sensitivity of the double wedge-shaped ionization chamber (IQM) to errors in segment using the BEAMnrc/EGSnrc Monte Carlo (MC) simulation. The input value in this case is the accelerator beam segment and the output is the corresponding dose scored in the double wedge ionization chamber of the IQM system.

## 2. Materials and Methods

### 2.1. Simulation Setup

**Figure 2:**
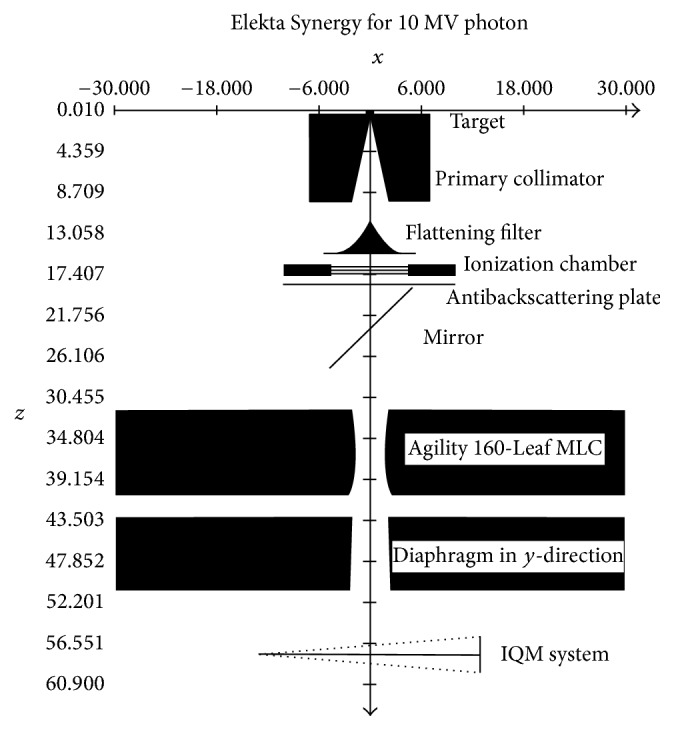
Setup of the IQM geometry located 4.5 cm below the *Y*-diaphragm for 10 × 10 cm^2^ field.

BEAMnrc MC was used to simulate an accurate source model of an Elekta Synergy linear accelerator equipped with an Agility 160-Leaf multileaf collimator (MLC) alongside the IQM. The IQM model was located 4.5 cm below the lowest diaphragm of the Linac model (Figure 2). The gradient of the IQM model is positioned perpendicularly to the MLC movement. During each simulation, the spatial integral dose was scored in the air region of the wedge-shaped ionization chamber of the IQM model.

**Figure 3:**
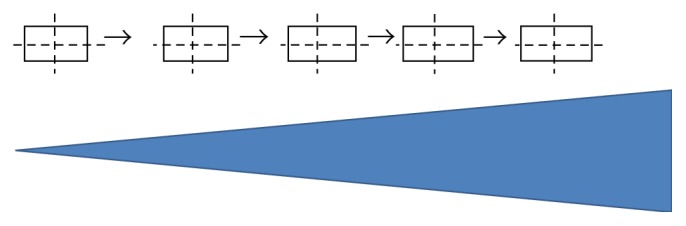
Movement of segment along the gradient of the IQM model.

To start the sensitivity study, regular fields of 3 × 3, 5 × 5, and 7 × 7 cm^2^ were simulated at 10 MV photon beams and moved along the gradient of the IQM model.  Figure 3 shows how a single segment was moved along the slope of the IQM. Dose responses were recorded along the gradient at positions of interest. The MLC defined field size remains unchanged along the IQM gradient, but the spot of the radiation beams on the IQM model was changed at every interval.

The sensitivity of the IQM was also studied by simulating six segments (regular and irregular) which were randomly altered within ±1, ±2, and ±3 mm of their original leaf positions to simulate leaf positional errors. The leaf positions were defined at the isocenter (100 cm SSD) and the beam energy studied was 10 MV. The regular and irregular segments (shown in Table 1) were chosen to include a wide range of MLC shaped aperture conditions. Each of the open leaves of the segments was randomly altered. The ±1 mm alteration means that any randomly generated value between −1 mm and +1 mm can be chosen to simulate a shift from the original leaf position. Note that there is a probability of having a zero value from the random generator, which means that the particular leaf will not be altered. For a segment, each positional error (±1, ±2, and ±3 mm) was altered 10 times to have a total of 30 altered models per segment. This procedure was repeated for each of the segments used in this study (Table 1). MC simulations were done for 180 altered and 6 unaltered segments, and the corresponding IQM signal responses were collected from each simulation for analysis.

The number of histories was large enough to reduce uncertainties in the scored IQM dose to less than 1%. The following simulation parameters were set: the global electron cut-off energy (ECUT) was set to 0.7 MeV and the global photon cut-off energy (PCUT) was set to 0.01 MeV for efficiency [28]. The maximum step size (SMAX) was set to default because EXACT was used in the boundary algorithm and PRESTA II was used in the electron-step algorithm. The maximum fraction energy loss/step (ESTEPE) was set to 0.25 (25%) and the maximum first elastic scattering moment per step (Xlmax) was set to 0.5. The skin depth for the boundary crossing algorithm (skindepth_for_bca) was set to 0 cm. Spin effects for electron elastic scattering (spin_effects) were turned on for appropriate backscattering simulation and electron impact ionization (eii_flag) was turned off. Bremsstrahlung angular sampling (IBRDST) was set to the default method and its cross sections were derived from the Bethe-Heitler method. Bound Compton scattering (IBCMP) was turned on and its cross section data were obtained from its default method. Pair angular sampling (IPRDST) was set to the Bethe-Heitler method (default). Photoelectron angular sampling (IPHTER) and Rayleigh scattering (IRAYLR) were turned off, and atomic relaxations (IEDGFL) were turned on. The photon cross-section data bases (photon_xsections) were set to the PEG4 dataset. All EGSnrc simulation parameters were conventionally set for a better reflection of realistic radiation transport [29].

### 2.2. Scatter Plots Sensitivity Analysis

Scatter plots (SP) can be used for qualitative analysis to determine the most sensitive parameter if more than one input variable is used that can alter the output result. It is achieved by plotting the graph of the input values against the corresponding output values. The linearity of the scatter plots determines the sensitivity of the model. An increase in the gradient of the linear equation indicates that the model is more sensitive to the input parameter under consideration [30, 31]. SP can be utilized to determine the IQM sensitivity by correlating the effect of segment area (SA) input on its output signal (*S*).

### 2.3. Brute Force Sensitivity Analysis

The brute force sensitivity analysis method is based on noninteractive input variables whereby the input variable is altered to study its effect on the output value. It generates the sensitivity of a model due to perturbation of the input variables [32]. Quantification of the input variables is necessary. As mentioned above, this study quantifies SA as the input parameter. A sensitivity index (SI) was calculated based on the change in *S* (Δ*S*) over the corresponding change in SA (ΔSA) [33–35]:

(1) $$\begin{array}{c} SI={\left|\;\frac{\Delta \left(S\right)}{\Delta \left(SA\right)}\;\right|}_{i}, \end{array}$$

where Δ(*S*) = *d*(*S* − *S*_*i*_)  *S* is the signal for the unaltered segment (SA) and *S*_*i*_ is the signal after segment alteration (SA_*i*_). Large SI indicates that the change in SA has a significant impact on *S*.

### 2.4. Variance-Based Sensitivity Analysis

The variance-based sensitivity analysis (VAR) method is a global sensitivity analysis; it is based on the idea that one can deduce the sensitivity of a model through its variance (*V*). It is a probability distribution of the output uncertainties (in this case, the output uncertainty is the uncertainty in *S*). It focuses on interactions between the input variables and the effect of each of the input variables on the output value. It is a measure of the importance of input variables on the outputs [22, 36, 37]. In our application, the sensitivity of the output to the input variable is therefore measured by the amount of variance in *S* caused by alterations in SA:

(2) $$\begin{array}{c} Y=f\left(\;X_{i}\;\right). \end{array}$$

In (2), *Y* represents *S* and *X*_*i*_ is the change in segment size generated at random for a given segment. It should be noted that only SA plays a role in the variation of *S* and thus index *i* = 1 in (2). If some other input variable also played a role, then index *i* = 1, 2.

The sensitivity measure of conditional variance *V*_*i*_ for a single input variable is given by

(3) $$\begin{array}{c} V_{i}=V\left(\;E\left[\;Y\;|\;X_{i}\;\right]\;\right). \end{array}$$

The sensitivity measure of sensitivity index SI_*i*_ is given by

(4) $$\begin{array}{c} SI_{i}=\frac{V_{i}}{V\left(Y\right)}, \end{array}$$

where *V*(*Y*) is the unconditional variance.

SI_*i*_ is the main effect index (first-order sensitivity index or correlation ratio) and it describes the main effect of the given data *X*_*i*_ on the value *Y* [17, 31, 38].

In this study, the effects of other input variables on the IQM output value are not present. Therefore, an extension for the total effect index is not applicable since the focus is on only one input variable.

### 2.5. Standard Regression Coefficient Sensitivity Analysis

This is a regression analysis that tests the significance of an independent variable (input) on its dependent variable (output) by using both the mean and the variance of the independent and dependent variables across the observable model. It is generally used for linear regression [22, 23].

The generalized linear regression model is

(5) $$\begin{array}{c} Y=b_{0}+\overset{n}{\underset{i=1}{\sum }}b_{i}X_{i}. \end{array}$$

The normalized regression model for an input variable is

(6) $$\begin{array}{c} \frac{Y_{i}-\bar{Y}}{s}=\left(\;\frac{b_{i}s_{i}}{s}\;\right)\frac{X_{i}-\bar{X}}{s_{i}}. \end{array}$$

The standard regression coefficient (SRC) SRC = *b*_*i*_*s*_*i*_/*s* and *b*_*i*_ is the regression coefficient for the *i*th sample of the *X* input (segment area), where

(7) s=1N−1∑i=1NYi−Y¯2,si=1N−1∑i=1NXi−X¯2

are the standard deviation of output signal (*Y*) of the IQM and segment area (*X*), respectively. The sensitivity of the model is determined by the SRC value. The higher the SRC value, the more sensitive the variation in the input value on the variation of the output value.

## 3. Results and Discussion

**Figure 4:**
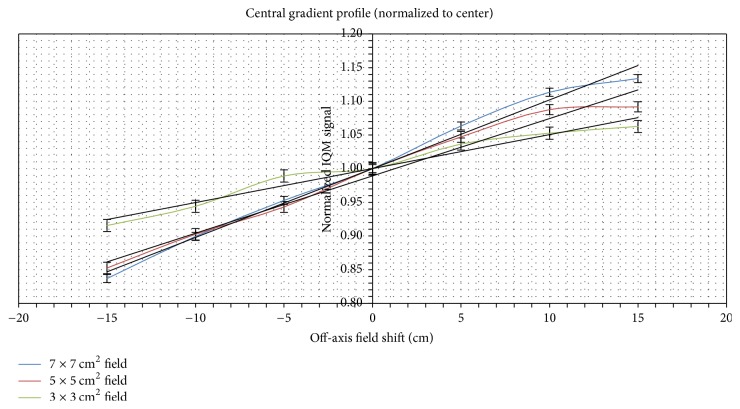
Sensitivity factor along the IQM CM gradient for 3 × 3, 5 × 5, and 7 × 7 cm^2^ fields (trend gradients are 0.005, 0.009, and 0.010).

Figure 4 shows the sensitivity gradient profiles for 3 × 3, 5 × 5, and 7 × 7 cm^2^ fields at 10 MV photon beams. This is to compare the gradient response between different segments. The sensitivity profile increases along the gradient of the IQM model. There is a noticeable plateau region in the sensitivity profile. This is the region of the higher separation distance between the collecting plate and the polarizing plate. It should also be noted that the photon beam of a field that is incident on the IQM model is not the same along the gradient of the IQM. The incident aperture changes along the gradient. The combined effect of nonuniform incident aperture and the separation distance between the plates gives the sensitivity profile. In Figure 4, the gradient of the trend line increases with an increase in field sizes.

In Figure 4, the largest air volume region has the highest signal response. Around the region of the largest air volume of the IQM chamber, there is a noticeable plateau. This plateau is caused by loss in lateral electron equilibrium (Farrokhkish's presentation also stated this idea at the World Medical Physics Conference (IUPESM 2015) [27]). Loss of lateral equilibrium could be a result of the photon beam being unable to completely cover the air volume of the IQM chamber at this region [39].

**Figure 5:**
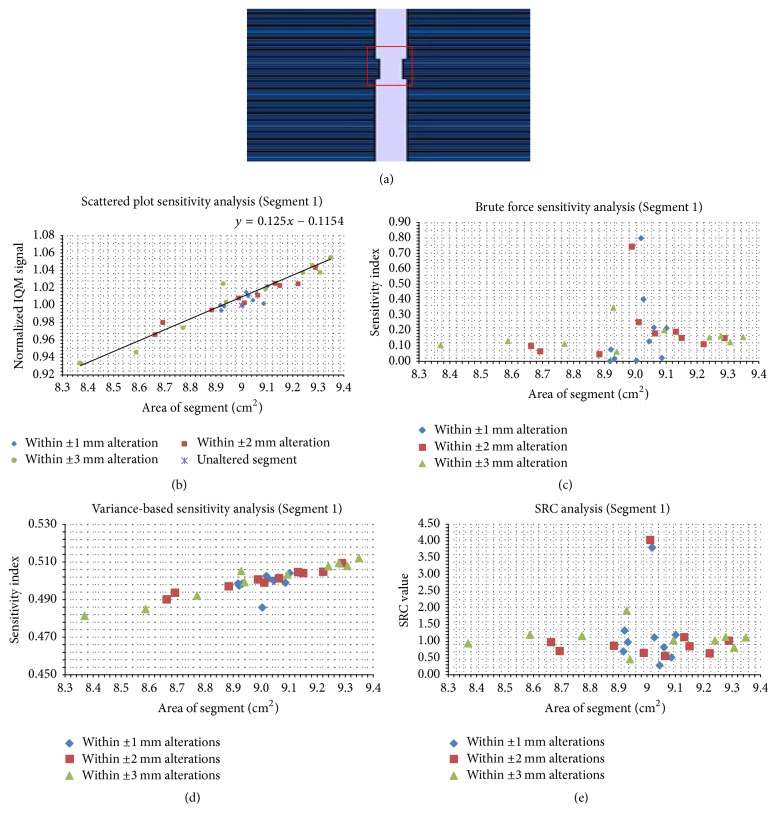
Sensitivity analysis results for the 3 × 3 cm^2^ segment. Panel (a) shows the unaltered segment; panel (b) shows the scatter plot; panels (c), (d), and (e) show the brute force, variance-based, and standard regression coefficient results, respectively.

**Figure 6:**
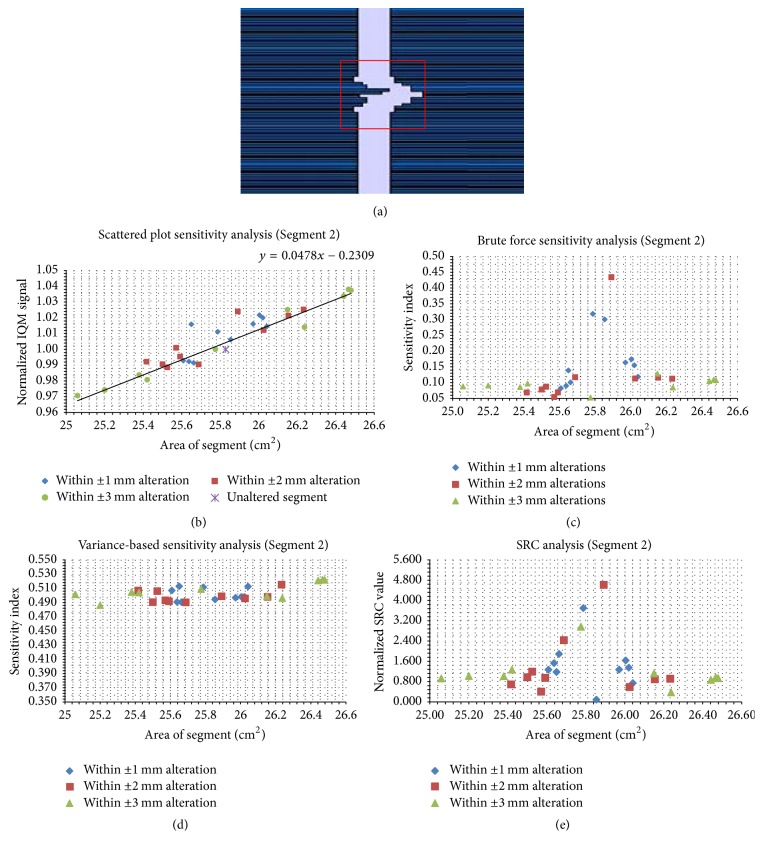
Sensitivity analysis results for an irregular segment (SA = 25.83 cm^2^). Panel (a) shows the unaltered segment; panel (b) shows the scatter plots; panels (c), (d), and (e) show the brute force, variance-based, and standard regression coefficient results, respectively.

**Figure 7:**
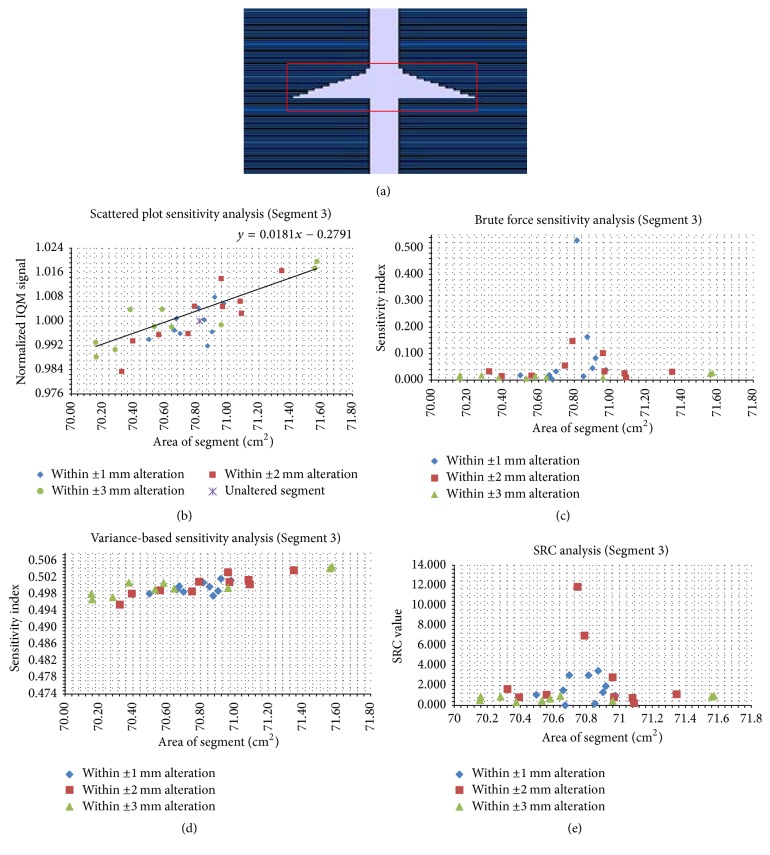
The same data as in Figure 6, but for the irregular segment with an area of 70.82 cm^2^. Panel (a) shows the unaltered segment; panel (b) shows the scatter plots; panels (c), (d), and (e) show the brute force, variance-based, and standard regression coefficient results, respectively.

**Figure 8:**
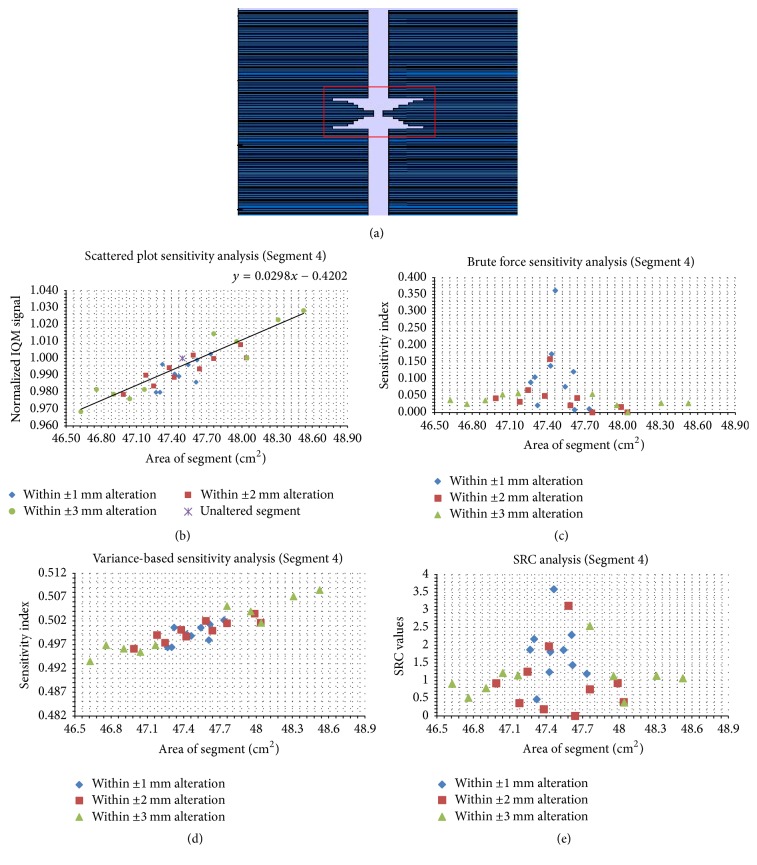
The same data as in Figure 4, but for the irregular segment with SA of 47.49 cm^2^. Panel (a) shows the unaltered segment; panel (b) shows the scatter plots; panels (c), (d), and (e) show the brute force, variance-based, and standard regression coefficient results, respectively.

**Figure 9:**
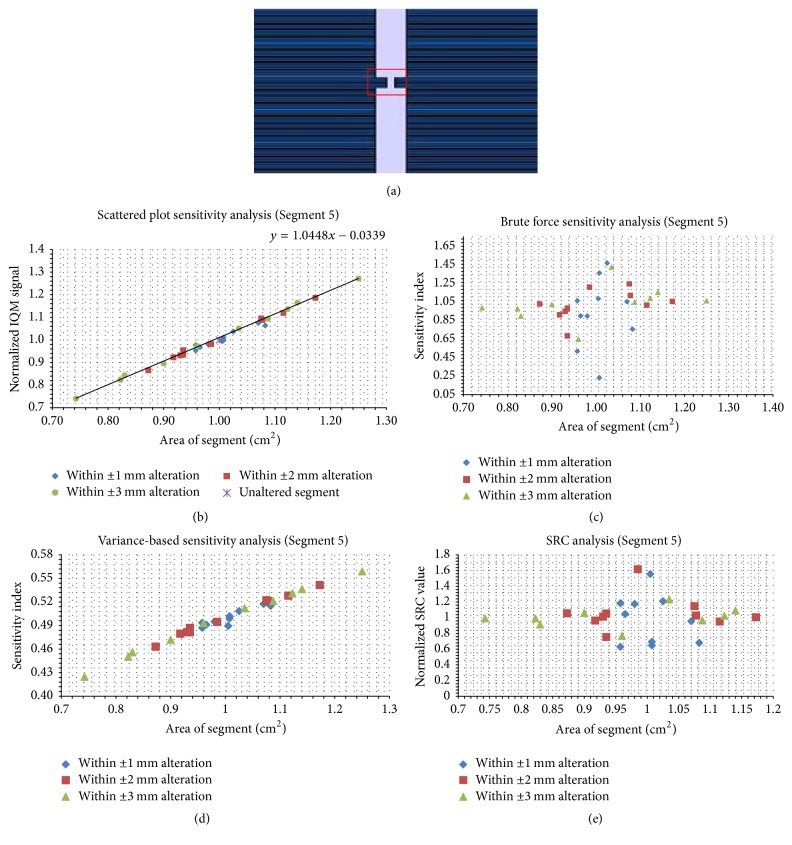
The same data as in Figure 4, but for the irregular segment with an area of 1.0 cm^2^. Panel (a) shows the unaltered segment; panel (b) shows the scatter plots; panels (c), (d), and (e) show the brute force, variance-based, and standard regression coefficient results, respectively.

**Figure 10:**
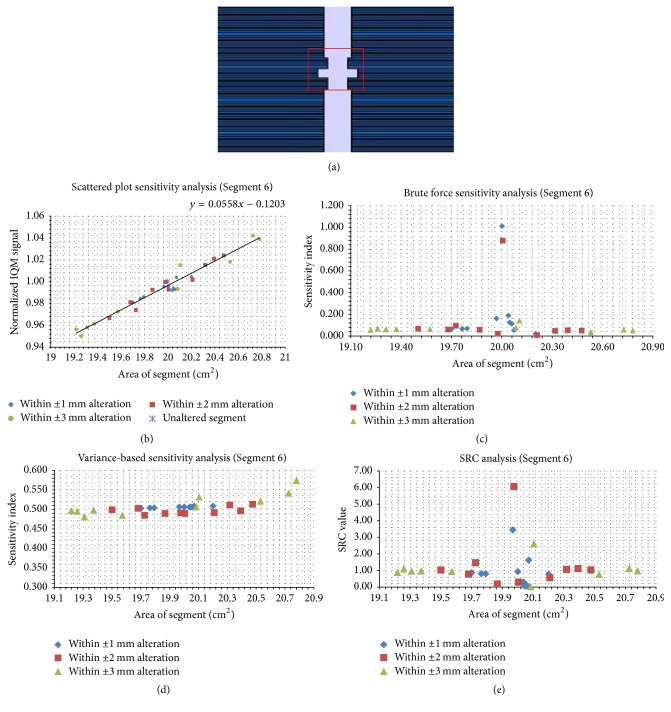
Sensitivity analysis results for an irregular segment (SA = 19.99 cm^2^). Panel (a) shows the unaltered segment; panel (b) shows the scatter plots; panels (c), (d), and (e) show the brute force, variance-based, and standard regression coefficient results, respectively.

Figures 5–10 depict five segments that were altered randomly within the limits stated above and their sensitivity analysis results.

In Figure 5(a), a regular 3 × 3 cm^2^ field is presented. In Figure 5(b), SP data are shown; for each maximum alteration level such as ±1 mm, there are 10 data points which correspond to the 10 trials that were measured as outlined above. Data for the ±2 mm and ±3 mm cases are also plotted in Figure 5(b). There exists a correlation between *S* and SA, indicating a definite response to the IQM signal when the segment area is altered. The SA range depends on the allowed segment alteration limit. For the ±3 mm randomly altered case, SA ranged between 8.38 cm^2^ and 9.36 cm^2^. Originally, SA was 9.00 cm^2^. For the ±2 mm case, this range shrank to between 8.66 and 9.29 cm^2^. For the ±1 mm case, there is an even spread around the unaltered SA. The linear trend line of SP has a gradient of 0.13 cm^−2^. In Figure 5(c), the brute force analysis tool shows the rate of change of *S* with respect to SA. Thirty SI values are shown indicating 30 trials. High SI values are noted around the unaltered SA. In Figure 5(d), SI values were analyzed for 30 trials using the VAR method. SI increased with increasing SA values. In Figure 5(e), the 30 normalized SRC values are depicted. High SRC values are seen around the unaltered SA.

The irregular segment in Figure 6(a) was altered within ±1, ±2, and ±3 mm. SP (Figure 6(b)) have a trend-line gradient of 0.048 cm^−2^ for the SA alterations that span the range of 25.10–26.46 cm^2^. In Figures 6(c) and 6(e), the brute force analysis and SRC values are displayed. The two methods show that the most sensitive altered segment is around the unaltered segment (SA = 25.80 cm^2^). In Figure 6(d), the VAR indices correspond to SA values for the 30 trials.

Figure 7(a) shows an irregular segment (SA = 70.86 cm^2^), the largest SA considered in this study. In Figure 7(b), IQM *S* shows a sensitivity to SA indicated with a gradient of 0.018 cm^−2^. In Figure 7(c), the brute force sensitivity indices show that higher sensitivity indices are noted around the unaltered segment. One of the alterations within ±1 mm has the highest sensitivity index of 0.52. In Figure 7(d), the variance of the IQM signal output determines the sensitivity of the IQM model by correlating the VAR sensitivity indices with 30 trials. An increase in SA causes an increase in VAR sensitivity index. In Figure 7(e), SRC were plotted against SA. The highest SRC value was observed in one of the alterations within ±2 mm. This is the most sensitive altered segment out of the 30 trials.

In Figure 8(a), the model of an irregular segment of 47.49 cm^2^ is presented. In Figure 8(b), SP values for 30 trials are shown. An increase in SA causes a gradual increase in *S*. The *S* values are narrowly scattered around the trend line with a gradient of 0.030 cm^−2^. In Figure 8(c), the brute force sensitivity indices are displayed for the trials considered. It is observed that the most sensitive indices are for small alterations of the original SA. In Figure 8(d), the VAR SI correlate with SA; an increase in SA causes an increase in SI. In Figure 8(e), the normalized SRC values were plotted against the 30 SAs considered. The maximum SRC value is observed around the region of the original SA.

In Figure 9(a), a regular segment of SA = 1 × 1 cm^2^ is shown. This is the smallest SA considered in this study. In Figure 9(b), the data points align with the linear trend line with a gradient of 1.045 cm^−2^. In Figures 9(c) and 9(e), the brute force analyses and SRC values show high sensitivities for all the trials considered with the highest SI around the original segment area. In Figure 7(d), the VAR SI shows a linear progression with an increase in segment areas of the 30 trials.

Figure 10(a) is an irregular segment that was altered within ±1, ±2, and ±3 mm. SP (Figure 10(b)) have a trend-line gradient of 0.06 cm^−2^ for the SA alterations that span the range of 19.21–20.79 cm^2^. In Figures 10(c) and 10(e), the brute force analysis and SRC values are displayed. The two methods show that the most sensitive altered segment is around the unaltered segment (SA = 19.99 cm^2^). In Figure 10(d), the VAR indices correspond to SA values for the 30 trials.

The highest degree of linearity in SP is found in the smallest SA of 1 × 1 cm^2^ (Figure 9(b)). For this SA, the SP values were almost on the trend line and it has the smallest gradient of 0.018 cm^−2^ (Figure 7(b)).

**Figure 11:**
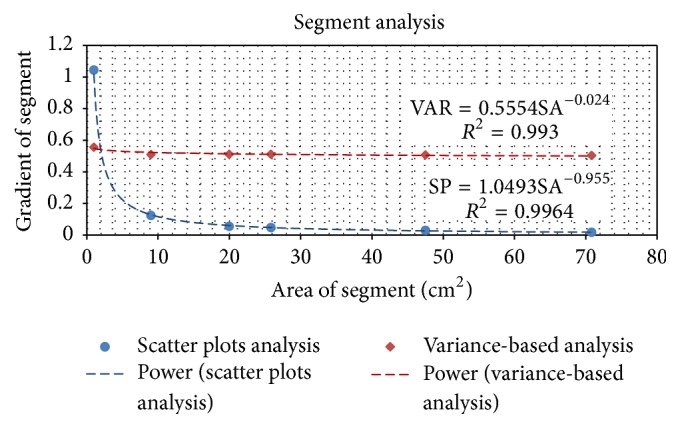
Analysis for scatter plots and variance-based sensitivity analysis for Segments 1–6.

In Figure 11, the gradient of scatter plots (SP) and variance-based (VAR) sensitivity analysis in Figures 5–10 were plotted. A power function was fitted to the SP trend-line gradient data as a function of original SA (SP = 1.0493SA^−0.955^). It shows that an increase in SA causes a decrease in the SP trend-line gradient that is at first strongly dependent on SA but becomes less sensitive for SA ≥ 26 cm^2^. This indicated that the IQM detects small shifts in the smallest segment with the largest differential signal which is to be expected since the allowed random shifts between ±1, ±2, and ±3 mm of the MLC leaves would make up a larger percentage change in SA compared to the largest segment; larger segments will cause smaller differential signals. SP data are a quick means of investigating the SI of data but do not compare the rate of change in IQM signals to the rate of change in SA like the brute force sensitivity analysis.

In Figure 11, the SP and VAR sensitivity analysis methods show power function relationships with respect to SA although the VAR is virtually constant over SA.

VAR indices for the five segments considered decrease weakly with an increase in SA and can be considered for practical purposes to be constant with an average value of 0.525. The fitted power function

(8) $$\begin{array}{c} VAR=0.555SA^{-0.024} \end{array}$$

has the derivative of

(9) $$\begin{array}{c} \frac{d\left(VAR\right)}{d\left(SA\right)}=\frac{0.013}{SA^{1.014}}. \end{array}$$

From (9), it is seen that the rate of change in VAR with change in SA is approximately inversely proportional to SA for the segment sizes studied. It has a maximum at the smallest segment and decreases as SA increases starting off at an initial value of 0.013.

Brute force sensitivity analysis for the segments in Figures 5(c), 6(c), 7(c), 8(c), and 10(c) shows that the highest SI is within the region of the unaltered (original) segments, while in the small field (Figure 9(c)) it is high for all trials considered. This again indicates that the IQM signal is sensitive to alterations of small segments and less sensitive to alterations of large ones. The brute force technique means that if a segment is altered, the magnitude of alteration (perturbation) which is the difference between the unaltered and altered SA must be evaluated based on the difference in the magnitude of *S*. If the minimal difference in SA gives a significant difference in *S*, then the model is very sensitive to such input parameter. This technique, unlike scatter plots, considers the rate of change of the altered parameters. If the SP is highly linear (Figure 9(b)), the brute force SI (Figure 9(b)) will display a noticeable deviation from zero across all altered segments.

SRC values for the five segments displayed maximum (normalized) values around the region of the unaltered segments. For the smallest SA (Figure 9(e)), higher SRC values were calculated across all 30 altered SA trials. SP data give a reasonable indication of this outcome. For a perfectly linear graph, the rate of change of the output value per the input variable will be uniform, which makes the SRC values seem uniform across the altered segments around original SA = 1 × 1 cm^2^. This means that the IQM is most sensitive to alterations of small SA.

In total, 6 original segments were each altered 30 times randomly within ±1, ±2, and ±3 mm positional errors. For each segment, a Monte Carlo simulation was done to determine the IQM signal. Sensitivity analysis results indicated that the IQM is sensitive to detect these alterations in SA. The sensitivity is more pronounced in small SA.

## 4. Conclusion

The sensitivity of the IQM in this study shows its potential to detect small alterations in SA. The SP and VAR relation to SA is approximately constant at large SA but displays power function relationships at smaller SA values. All sensitivity analysis methods employed in this study indicated that the IQM signal (*S*) will indicate small segments alterations even for the larger segments.

## Figures and Tables

**Table 1:** List of segments altered randomly within ±1, ±2, and ±3 mm.

Segments	Segment area (cm^2^)
Segment 1 (3 × 3 cm^2^)	9.00
Segment 2 (irregular)	25.83
Segment 3 (irregular)	70.82
Segment 4 (irregular)	47.49
Segment 5 (1 × 1 cm^2^)	1.00
Segment 6 (irregular)	19.99
